# Patterned Superhydrophobic SERS Substrates for Sample Pre-Concentration and Demonstration of Its Utility through Monitoring of Inhibitory Effects of Paraoxon and Carbaryl on AChE

**DOI:** 10.3390/molecules25092223

**Published:** 2020-05-08

**Authors:** Umi Yamaguchi, Maki Ogawa, Hiroyuki Takei

**Affiliations:** 1 Graduate School of Life Sciences, Toyo University, Itakura, Gunma 374-0193, Japan; umily1116@gmail.com; 2 Faculty of Life Sciences, Toyo University, Itakura, Gunma 374-0193, Japan; ogawa.maki.t1@dc.tohoku.ac.jp; 3 Bio Nano Electronics Research Centre, Toyo University, Kawagoe, Saitama 350-0815, Japan

**Keywords:** SERS, superhydrophobicity, pre-concentration, AChE, inhibitor

## Abstract

We describe a patterned surface-enhanced Raman spectroscopy (SERS) substrate with the ability to pre-concentrate target molecules. A surface-adsorbed nanosphere monolayer can serve two different functions. First, it can be made into a SERS platform when covered by silver. Alternatively, it can be fashioned into a superhydrophobic surface when coated with a hydrophobic molecular species such as decyltrimethoxy silane (DCTMS). Thus, if silver is patterned onto a latter type of substrate, a SERS spot surrounded by a superhydrophobic surface can be prepared. When an aqueous sample is placed on it and allowed to dry, target molecules in the sample become pre-concentrated. We demonstrate the utility of the patterned SERS substrate by evaluating the effects of inhibitors to acetylcholinesterase (AChE). AChE is a popular target for drugs and pesticides because it plays a critical role in nerve signal transduction. We monitored the enzymatic activity of AChE through the SERS spectrum of thiocholine (TC), the end product from acetylthiocholine (ATC). Inhibitory effects of paraoxon and carbaryl on AChE were evaluated from the TC peak intensity. We show that the patterned SERS substrate can reduce both the necessary volumes and concentrations of the enzyme and substrate by a few orders of magnitude in comparison to a non-patterned SERS substrate and the conventional colorimetric method.

## 1. Introduction

Surface-enhanced Raman spectroscopy (SERS) has come a long way since its discovery in the 1970s [1,2]. It has become a powerful analytical tool in basic sciences and industry for the identification of chemical species. It has sufficient sensitivity to detect a single molecule [3], and there are an increasing number of reports on its applications in wide-ranging fields [4,5]. The past decade has seen increasing offerings of commercial SERS materials and substrate [6,7]. SERS has the obvious advantage of significantly enhancing the scattering intensity, but this also has a positive effect on the hardware development because low intensity light sources are sufficient for obtaining SERS spectra, allowing the development of compact and less expensive instruments [8,9]. As instruments dedicated to SERS become more widely available and prices of substrates come down due to increased production, hurdles for carrying out SERS analysis should become lower.

SERS can be applied to the detection of various target molecules, but it is particularly in the field of biosensing that is receiving much attention [10,11,12]. Target molecules range from antibiotics to pesticides, food additives, pathogens, and opioids, etc. [9,13,14,15,16,17,18,19,20]. There are many approaches to carrying out SERS measurements and the simplest and most widely reported method makes use of colloids. Noble metal nanoparticles such as colloids generate a strong near-field when free electrons are induced to oscillate resonantly by an impinging electromagnetic field [21]. This property of noble metal colloids has been used in various analytical techniques including SERS [22]. When colloids are mixed with the target molecule, a SERS spectrum of the target can be obtained. This approach is sufficient for many applications, but there are a number of disadvantages. The enhancement effect depends on the concentration as well as the aggregation state of colloids. This means that the dilution of the target molecule solution upon mixing with the colloidal solution and the extent of aggregation affected by ionic strength need to be carefully taken into account for quantitative measurements.

A smaller number of groups have made use of surface-adsorbed systems, either with colloids adsorbed on a substrate or metal nanostructures directly fabricated on a substrate [6,7]. Substrates are more convenient to use because a liquid sample can be simply placed on a substrate and an excess sample can be readily washed off. Another unique aspect of a substrate system is the ability to pre-concentrate target molecules prior to SERS measurements [23]. This is accomplished by preparing a superhydrophobic SERS substrate. When an aqueous sample is applied to such a substrate, the sample takes the shape of a sphere. Subsequent drying leads to pre-concentration of the target molecule onto a reduced area. Li et al. constructed a silver nanowire platform whereby a layer of silver nanowires was prepared by the Langmuir–Schaefer techniques, and a multiple number of such layers, each rotated 90 degrees with respect to the previous layer, were stacked up for the preparation of a SERS substrate. Treatment of the substrate by perfluorodecanethiol (PFDT) made the surface superhydrophobic. With the contact angle approaching 150 degrees, they reported significant improvements in sensitivity [24]. Sepaniac et al. has also made use of a superhydrophobic SERS surface for improving the sensitivity [25]. Hakonen et al. described a hydrophobic nanopillar system capable of detecting picric acid on the order of 20 ppt [26]. Deng et al. reported on a hierarchical superhydrophobic system consisting of a layer of 4.24 µm melamine-formaldehyde spheres coated with 43.8 nm Ag nanoparticles. Treatment of such a structure with PFDT resulted in a superhydrophobic surface with a contact angle approaching 150 degrees. They reported improved detection sensitivities toward dopamine, lysozyme and hemoglobin [27]. Wang et al. coated paper with Ag nanodendrites, again with the goal of preparing a superhydrophobic SERS substrate [28].

In this paper, we describe an alternative approach for preparing superhydrophobic SERS substrates. Our intension is to achieve improvements in three areas. For one, we are interested in controlling the extent of pre-concentration. When an aqueous sample is placed on a uniformly hydrophobic surface, the actual area of contact on the substrate cannot be precisely controlled. The system we discuss here has a SERS spot with a precise dimension surrounded by a superhydrophobic area. This allows us to control the extent of pre-concentration. The second aspect of improvement is related to the placing of an aqueous sample. When the entire surface is superhydrophobic, it is difficult to transfer a small-volume sample droplet onto the surface from an application aid such as a pipette because a droplet tends to cling to the pipette tip rather than to the SERS surface. A SERS spot that is rendered less hydrophobic facilitates the transfer process greatly, and once the sample solution is on the substrate, it helps to anchor the sample at one location. Thirdly, our SERS surface is not coated with a chemical species for surface-chemical modification. While previous workers took the precaution of using a chemical species with minimum Raman activity, it is preferable to have no chemical species to start with. We achieved our goal in the following fashion. Our SERS substrate is based on a form of metal film on nanosphere (MFON). Most workers in this area have made use of a monolayer of highly monodisperse nanosphere in a well-ordered array format [29,30,31,32]. While MFONs have been used in many applications in plasmonics, they were ultimately derived from an earlier structure called nanosphere lithography (NSL) [33]. This required a regular array of nanospheres that needs to function as a shadow mask. We, on the other hand, make use of a monolayer of randomly-adsorbed nanospheres as a template for metal deposition [34]. There are two advantages with random MFON. For one, the adsorption process is significantly simplified because no regular array needs to be formed. With our protocol, the adsorption process is complete within tens of seconds, and substrates with surface areas much larger than dozens of cm^2^ can be readily prepared. Secondly, nanospheres do not have to be highly monodisperse. Moreover, there is no reduction in the performance of resulting SERS substrates. On the contrary, a slight reduction in the adsorption density or judicious mixing of nanospheres of different sizes can enhance the performance [35]. Random MFONs have been applied to various plasmonic applications such as localized surface plasmon resonance sensing [36] and metal-enhanced fluorescence [37] and combinations thereof [38] in addition to SERS.

The randomly-adsorbed nanosphere monolayer has another interesting property. It is well known that the contact angle of a rough hydrophobic surface is significantly increased over that of a smooth surface [39]. Thus, the presence of a nanosphere monolayer makes a water-repelling treatment more effective, rendering the surface superhydrophobic. If such a surface is patterned with silver by vacuum deposition, the area covered by silver turns into a SERS spot, as shown in Figure S1. Incidentally, the SERS spot is less hydrophobic than the surrounding non-metalized areas so that an aqueous sample applied with a pipette can be readily transferred onto the SERS spot. The diameter of the SERS spot determines the extent of pre-concentration.

We applied this patterned SERS system to a study of AChE [40], whose inhibitions have important environmental and medical relevance. One class of inhibitor molecules are pesticides. They have been studied by SERS [41]. While pesticides are indispensable part of the modern society, excessive applications or use of unsanctioned products can have disastrous results. Pesticides can be detected by numerous techniques such as the electrochemical method, gas chromatography and high-performance liquid chromatography. In recent years, the potential of SERS has become widely recognized as a simple and rapid method for pesticide detection [42]. Some pesticides can be directly detected as a SERS-active molecular species. A review article by Dias Soares and De Olivieira reported that phosmet, methyl parathion, malathion, fenthion, methamidophos, glyphosate, and paraoxon, among others, had been detected by SERS [43]. These chemicals belong to the class of organophosphorous pesticides. AChE becomes inhibited when a deeply buried active site becomes occupied by an inhibitor. The size of the active site of AChE is three times greater than other ester hydrolysis enzymes, bestowing it with marked efficiency [44]. Besides direct detection, some workers have investigated the possibility of monitoring the inhibitory effect on AChE by monitoring the formation of the enzymatic product by SERS [45,46]. It is fortuitous that the protocol by Ellman et al. for the traditional colorimetric method calls for the use of ATC, analog to naturally relevant acetylcholine, as the substrate [47]. ATC is hydrolyzed into acetic acid and thiocholine (TC). An S-H group of TC promotes its binding to a noble metal surface. Liron et al. were one of the first groups to report on the use of SERS for monitoring the formation of TC and the effect of adding an inhibitor [45]. They used a colloidal system to monitor the 772 cm^−1^ band of TC and showed that this band was suppressed upon addition of paraoxon, with the LOD of 1.8 × 10^−8^ M. Alami et al. also studied the effects of paraoxon and carbaryl by SERS, albeit through peaks associated with acetylcholine and choline [46]. They reported that the lowest detectable levels for paraoxon and carbaryl were 4.0 × 10^−14^ and 1.9 × 10^−9^ M, respectively. More recently, there were also reports on a similar colloid/acetylcholine system with the additional emphasis on the state of colloid aggregation [48] and a cautionary note on the effect of silver nanoparticles on hydrolysis [49]. A closely related enzyme, butyrylcholinesterase, was also investigated with SERS using silver paste [50].

In this paper, we first show results of evaluating the water-repelling property of various surfaces; a plane glass slide and a glass slide covered by a nanosphere monolayer, all of which with or without decyltrimethoxy silane (DCTMS) treatment. Contact angles of an aqueous droplet on these surfaces were measured. We then show how the SERS spot diameter affects the extent of pre-concentration; in one approach, we use a simple geometrical model to calculate the ratio between the sample volume and SERS spot area. We also show the experimental results of attempting to place a maximum amount of sample on a SERS spot of a particular size. Then, we show the results of taking the SERS spectrum of a model compound, 1,2-bis(4-pyridyl) ethylene (BPE), demonstrating that the signal intensity can be enhanced significantly and how the spot diameter affects it. In the second half of the paper, we discuss the detection of TC, the end product of ATC hydrolyzed by AChE. By using the CH_3_ rocking peak at 786 cm^−1^ of TC as the indicator for the AChE enzymatic activity, we show how we optimized concentrations of AChE and ACT, making sure that non-enzymatic break down of ACT is minimized. The amount of TC produced should not saturate the SERS surface; just enough TC should be produced so that the effects of the inhibitors, paraoxon and carbaryl, can be detected. We monitored the AChE activity, with and without an inhibitor, with both standard and patterned SERS substrates. We show that both types of SERS substrates are capable of monitoring the AChE activity. We show that less AChE and ATC at lower concentrations were necessary than with the conventional colorimetric assay. The patterned SERS substrate is particularly superior and the reduction in volume is three orders of magnitude, and the concentrations can be nine orders of magnitude less for AChE.

## 2. Results and Discussions

**Figure 1:**
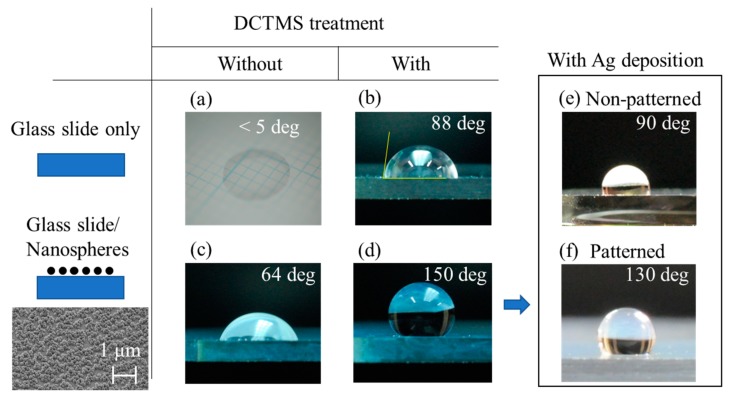
Photos of 1 μL water droplets placed on various surfaces. (**a**) plane glass slide as delivered, (**b**) glass slide treated with decyltrimethoxy silane (DCTMS), (**c**) glass slide covered by a monolayer of silica nanospheres, (**d**) glass slide covered by a monolayer of silica nanospheres after the DCTMS treatment, and (**e**) the substrate from (**d**) after deposition of 100 nm silver. The contact angle was the smallest, less than 5 degrees, for (**a**) and the largest at 150 degrees for (**d**). By depositing silver on the monolayer of DCTMS treated silica nanospheres, the surface became less hydrophobic (**e**), but at the boundary between the areas with/without silver deposition, the contact angle was 130 degrees (**f**).

First, we show results of contact angle measurements of a plane glass slide and a glass slide covered by a monolayer of silica nanospheres, with and without DCTMS treatment. The photos in Figure 1 show 1 μL water droplets placed on various surfaces; (a) a plane glass slide as delivered, (b) a glass slide treated with DCTMS, (c) a glass slide covered by a monolayer of silica nanospheres, (d) a glass slide covered by a monolayer of silica nanospheres and subjected to DCTMS treatment, and (e) the substrate from (d) after deposition of 100 nm silver. It can be seen that both DCTMS treatment and introduction of a monolayer of silica nanospheres leads to a significant increase in the contact angle from less than 5 degrees for a plane glass slide to 88 and 64 degrees, respectively. Combining both further increased the contact angle to 150 degrees (d). Additional deposition of 100 nm silver resulted in two contrasting ways depending on the area of deposition. With uniform deposition, the contact angle diminished significantly to 90 degrees, making it relatively more hydrophilic (e), but if the deposition area was limited by the use of a pin hole mask, the contact angle was 130 degree at the boundary between the areas with/without silver deposition (f). Here, a maximum volume of water was added without spilling outside the SERS spot. At the same time, the silver deposition makes the surface locally more hydrophilic so that an aqueous droplet can be more readily transferred onto it. If the entire surface was uniformly hydrophobic, transfer would be more challenging for small-volume samples of less than a few μL. Video clips in the Supplementary Information Section show attempts to place a 1 μL aqueous droplet onto a uniformly superhydrophobic area (Video1 (Superhydrophobic)) and the SERS spot of a patterned substrate (Video2 (SERSSpot)).

**Figure 2:**
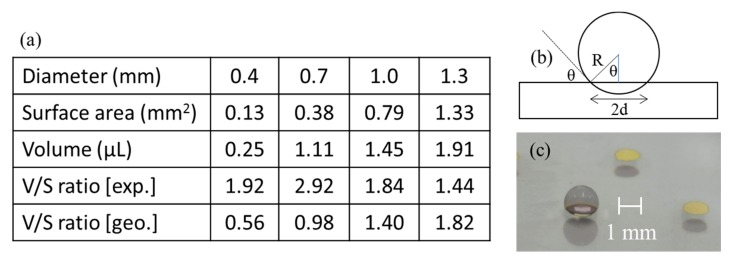
(**a**) Table showing surface-enhanced Raman spectroscopy (SERS) spot surface areas, maximum volumes of samples that can be placed, experimentally obtained and geometrically derived sample volume to the spot surface area ratio (volume/surface) for spot diameters of 0.4, 0.7, 1.0 and 1.3 mm with a simple geometric model (**b**). (**c**) Photo showing a 1.5 μL water droplet placed on a 1.0 mm SERS spot.

Next, we explored the influence of the SERS spot diameter on the extent of pre-concentration. We selected spot diameters of 0.4, 0.7, 1.0 and 1.3 mm to see if there was an optimal size. When one considers a perfect sphere as the shape of an aqueous droplet, the volume increases with the third power of the radius while the surface area increases only with the power squared. This means that larger radii might be preferable for more effective pre-concentration because of greater ratios between the sample volume and the contact area. However, larger volumes might result in the flattening of the sample droplet under the influence of the gravity. This would reduce the maximum sample volume that a SERS spot of a particular surface area can support without spilling over. Figure 2 includes a table showing SERS spot surface areas, the maximum volumes of a sample that can be placed, experimentally obtained and geometrically derived sample volume to spot surface area ratios (volume/surface ratio) and geometrically derived volume/surface area ratios for spot diameters of 0.4, 0.7, 1.0 and 1.3 mm. The last figure is obtained with a simple model (b) shown to the right of the table with an equation below.

(4πR^3^/3)/πd^2^ = 4 d/3 (sin θ)^3^ (1)

with the radius R of the spherical sample and the radius d of the SERS spot. θ is equal to the difference between 180 degrees and the contact angle. With the contact angle of 130 degrees obtained experimentally, the above ratio is approximately 2.81 d. Photo (c) in Figure 2 shows a 1.5 μL water droplet on a 1.0 mm SERS spot, one taken from an oblique angle to show both the water droplet and the SERS spot. All SERS spots in this photo are of the same diameter, even though the one with a water droplet on top looks slightly smaller. Photo (f) in Figure 1 would be its side view.

In contrast to the geometrically obtained volume/surface area ratios, the corresponding experimental ratios were not necessarily greater for larger SERS spots. This was caused by the difficulty in confining the water droplet within the SERS spot area, and we decided to be conservative in judging the maximum sample volume that could be placed. This suggests that a further increase in the SERS spot size was not recommended. The minimum size for the SERS spot size was dictated by our ability to handle aqueous droplets. Quantification was more difficult, and the transfer of the droplet from the tip of a pipette to the SERS spot became more challenging for smaller spot diameters. Further reduction in volume would need improvements in these areas. Experimentally, the spot diameter of 1.0 mm turned out to be practical.

With Figure S2, we show the level of uniformity one can expect from our AgFON. The uniformity of SERS signals depends on the uniformities of (1) nanostructures and (2) absorption of the target molecules. To separate these two effects and show only the former, we exposed our AgFON to a vapor of methyl mercaptan; alkanethiols are known to form a self-assembled monolayer, thus desirable as a standard model target for the evaluation of nanostructure uniformity. The intensity of the ν(C-S) peak at 678 cm^−1^ was used as a benchmark. Three separate substrates were prepared and spectra were obtained from five randomly chosen spots for each substrate. Figure S2a is a superposition of five spectra from one such substrate. The 678 cm^−1^ peak was baseline-corrected (Figure S2b). The averaged peak intensities are shown for three substrates in Figure S2c).

**Figure 3:**
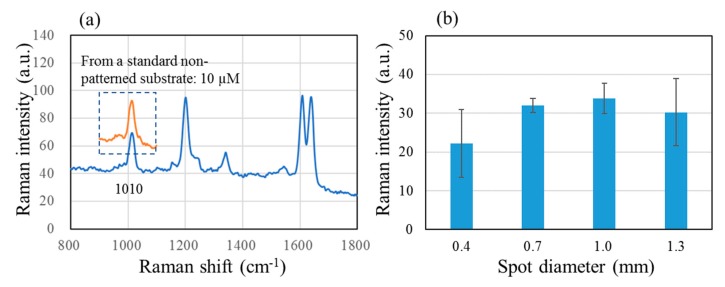
(**a**) SERS spectrum of 0.01 µM 1,2-bis(4-pyridyl) ethylene (BPE) obtained after pre-concentration on the patterned SERS substrate with 0.7 mm spot, superimposed with the 1010 cm^−1^ peak for 10 μM BPE obtained with a standard non-patterned substrate and (**b**) a bar graph of the 1010 cm^−1^ peak intensity with spot diameters of 0.4, 0.7, 1.0 and 1.3 mm.

Next, we explored whether pre-concentration would actually translate into greater SERS signals by using a Raman active molecule, trans-1,2-bis(4-pyridyl) ethylene (BPE) in solution. Figure 3 shows (a) a SERS spectrum of 0.01 µM BPE after pre-concentration with a 0.7 mm SERS spot and (b) a bar graph showing the intensity of 1010 cm^−1^ peak obtained with spot diameters of 0.4, 0.7, 1.0 and 1.3 mm. The 1010 cm^−1^ peak obtained from a standard non-patterned SERS substrate is superimposed onto the spectrum in (a); the BPE concentration had to be 1000 times greater at 10 µM to give a peak with a similar height. The bar graph (b) seems to indicate that there is little dependence on the spot diameter, but one must be careful to consider that the peak intensity does not depend linearly on the sample concentration. Figure S3 shows this dependence whereby standard non-patterned SERS substrates were immersed in BPE solutions for five minutes and subsequently blow-dried. Peak intensities of 22.2, 32, 33.8 and 30.27 correspond to BPE concentrations of 4.5, 17.5, 21 and 14.5 µM. This means, for example, that to generate a peak whose intensity is 33.8 rather than 22.2, the effective BPE concentration needs to be greater by the ratio between 21 and 4.5 µM, or 4.67. Thus, effective pre-concentration must be greater by 4.67. In addition, when one consider the standard deviation, the spot sizes of 0.7 or 1.0 mm are preferable to those of 0.4 or 1.3 mm.

Next, we show how we devised our protocol for carrying out the AChE assay with a standard non-patterned SERS surface. Elman et al. established the standard protocol for detection of TC produced by AChE [47], but we had to modify the protocol for SERS measurements. One must recognize one major difference between the conventional colorimetric method and the SERS approach. With the former, one measures the total amount of TC suspended in a liquid phase, but with SERS, one monitors the amount of TC that adsorbs onto the SERS substrate. It is also important to sufficiently reduce the amount of TC produced. If an excess amount of TC saturates the SERS surface, the addition of an inhibitor is not likely to reduce the TC peak intensity.

**Figure 4:**
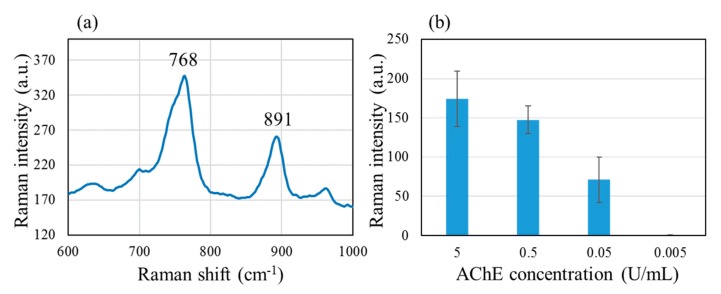
(**a**) SERS spectrum showing characteristic thiocholine (TC) peaks at 768 and 891 cm^−1^, obtained with the standard non-patterned SERS substrate. These peaks correspond to CH_3_ rocking and C-S stretching vibrations. (**b**) A bar graph showing the intensity of the 768 cm^−1^ peak monitored for various AChE concentrations, using 10^−4^ M ATC.

Figure 4 shows (a) characteristic TC peaks at 768 and 891 cm^−1^, obtained with the standard non-patterned SERS substrate. These peaks correspond to CH_3_ rocking and C-S stretching vibrations [45]. The more intense 768 cm^−1^ peak was chosen as the indicator for TC. The bar graph (b) shows that with 10^−4^ M ATC, the intensity of the 768 cm^−1^ peak shows gradually diminishing dependence on the AChE concentration in the range of 5 to 0.005 U/mL.

The results for higher concentrations of ATC, 10^−1^, 10^−2^ and 10^−3^ M, are shown in Figure S4. With 10^−1^ M ATC, no dependence on the AChE concentration could be observed. This is most likely due to the non-enzymatic breakdown of ATC [47]. With 10^−2^ and 10^−3^ M, dependence of the AChE concentration was observed, but the 768 cm^−1^ peak did not disappear even for the AChE concentration of 0.005 U/mL. We were concerned about the possibility of a catalytic activity of silver nanoparticles for ATC breakdown [46]. However, no 768 cm^−1^ peak was observed with the AChE concentration of 0.005 U/mL and the ATC concentration of 10^−4^ M, as can be seen in Figure 4b. We conclude that our standard non-patterned SERS substrate allows us to monitor the AChE activity with the AChE concentration down to 0.05 U/mL in combination with 10^−4^ M ATC. To monitor the effect by inhibitors, it is desirable that the amount of TC does not saturate the SERS spot surface. AChE concentration of 0.5 U/mL is a reasonable value because 5 U/mL would be close to saturation, and 0.05 U/mL would be closer to the detection limit.

**Figure 5:**
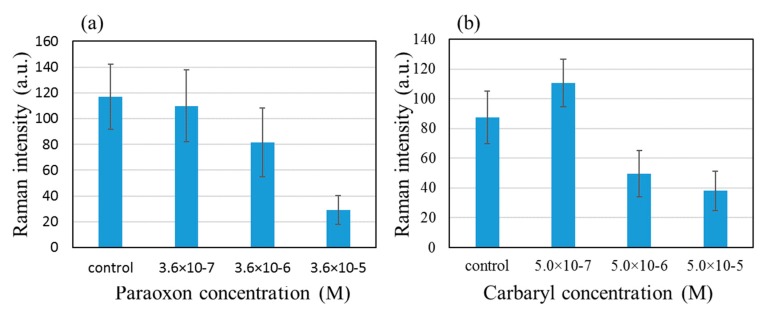
Loss in the intensity of the 768 cm^−1^ peak with addition of increasingly higher concentrations of (**a**) paraoxon and (**b**) carbaryl.

Now the effects of two inhibitors, paraoxon and carbaryl, were observed using the above condition, 10^−4^ M ATC and 0.05 U/mL AChE. Figure 5 shows that the 768 cm^−1^ peak intensity diminishes as paraoxon and carbaryl were added in increasingly higher concentrations, 3.6 × 10^−7^ to 3.6 × 10^−5^ M for paraoxon and 5.0 × 10^−7^ to 5.0 × 10^−5^ M for carbaryl.

Next, we used the above protocol as a basis for an adaptation to measurements with the patterned SERS substrate. One major difference from the above protocol is an additional step for drying the sample droplet. After equal volumes (5 μL) of ATC and AChE were mixed and allowed to react for 5 min, 1 μL of the mixture was placed on the SERS spot, which was immediately placed on a hot plate maintained at 90 °C. Heating served two purposes, (1) rapid drying, and (2) immediate termination of the enzymatic activity to obtain quantitative results.

**Figure 6:**
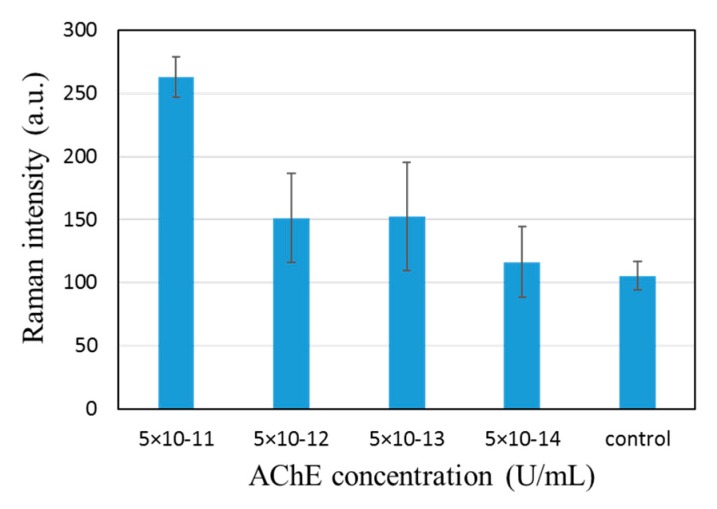
Bar graph showing the effect of pre-concentration on the intensity of the 768 cm^−1^ peak and its dependence on the AChE concentration. The peak can be observed even with AChE concentration of 5 × 10^−13^ U/mL with 10^−4^ M ATC.

We found that the AChE concentration could be reduced by many orders of magnitude, reaching 10^11^. The 768 cm^−1^ peak could be observed even with 5 × 10^−13^ U/mL AChE solution in combination with 10^−4^ M ATC, as shown in Figure 6. We have by no means completely optimized the measurement condition, but comparison with data in Figure 4 clearly shows a significant reduction in the required AChE concentration. The role played by the patterned SERS substrate is two-fold. For one, the reduction in the surface area of the SERS spot plays a role, estimated to be two-orders of magnitude. Second, pre-concentration allows a more even distribution of target molecules after complete drying. This allows us to capture all TC produced. With the standard non-patterned SERS substrate, however, we had to blow-dry the sample after a certain incubation time because controlling the area of contact between the substrate and aqueous sample was difficult. This implies that most TC produced would not be captured by the SERS surface.

For further comparison, we also carried out conventional colorimetric assays to demonstrate how much reduction in reagent volumes and concentrations is possible. The results are shown in Figure S5, showing that with 10^−4^ M ATC, the absorbance was as little as 0.18 with 0.005 U/mL AChE. The required ATC and AChE concentrations did not differ greatly from those for the standard non-patterned SERS substrate, but the required sample volume for the colorimetric assay was three orders of magnitude greater at 800 µL.

For assays with the patterned SERS substrate, we dried the sample by heating. As mentioned earlier, its purpose is two-fold—rapid drying and termination of the enzymatic activity. To confirm whether 90 °C was appropriate, we evaluated the effect of heating on the enzyme activity of AChE with both SERS and colorimetric assays, as shown in Figure S6. AChE solution in a tube was placed in a water bath at various temperatures for five min. Elevating the temperature to 60 °C clearly suppressed the AChE activity, and beyond 80 °C, the remaining signal is considered as a background.

**Figure 7:**
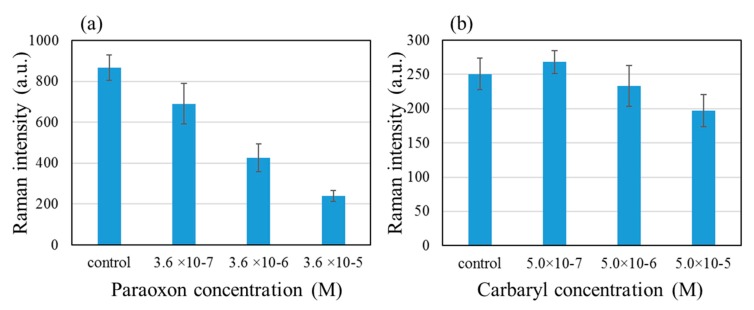
Inhibitory effects of (**a**) paraoxon and (**b**) carbaryl on the 768 cm^−1^ peak intensity of TC.

Finally, we show the results of evaluating the effects of paraoxon and carbaryl on the AChE activity using the patterned SERS substrate. The ATC and AChE concentrations were 10^−5^ M and 5 × 10^−8^ U/mL respectively. We explored the concentration range of 10^−7^ and 10^−4^ M for both inhibitors, and the 768 cm^−1^ peak intensity as a function of the inhibitor concentration is shown in Figure 7. The inhibitory effect was more clearly observed with paraoxon than with carbaryl. This is thought to reflect the irreversible nature of paraoxon binding to AChE vs the reversible nature of carbaryl-AChE binding.

## 3. Materials and Methods

Acetylcholinesterase (AChE, Cat. No. SLBV7012) and acetylthiocholine chloride (ATC, Cat. No. TBCBS6204V) were purchased from Sigma-Aldrich Co. LLC (St. Louis, MO, USA). For dilution, Tris HCl buffer (Cat. No. 24082-1, Polysciences Inc., Warrington, PA, USA) and distilled water (Cat. No. W-20 Trusco, Tokyo, Japan were used. We purchased 1 μg/μL methyl mercaptan/benzene standard solution (Cat. No. B0-06173), paraoxon (Cat. No. P-535) and carbaryl (Cat. No. M-632-03) from Fujifilm Wako Pure Chemical Co (Osaka, Japan). AChE was diluted with Tis HCl buffer to prepare 5, 0.5, 0.05 and 0.005 U/mL solutions. Acetylthiocholine chloride was diluted with distilled water to produce 10^−1^, 10^−2^, 10^−3^ and 10^−4^ M solutions. Paraoxon (M. W. 275) was diluted with distilled water to prepare 3.6 × 10^−5^, 3.6 × 10^−6^ and 3.6 × 10^−7^ M solutions. Carbaryl (M.W. 201) was diluted with distilled water to prepare 5.0 × 10^−5^, 5.0 × 10^−6^ and 5.0 × 10^−7^ M solutions.

Our SERS substrates were prepared as follows. A glass slide (Cat. No. SF17399, Matsunami Glass Ind., Ltd., Osaka, Japan) was treated with 3-aminopropyl trimethoxy silane (APTMS) by immersion in a 1vol%APTMS solution for 5 min. This treatment was necessary to promote the adsorption of silica nanospheres in the subsequent step. Exposure of the treated glass slide to a 50 mg/mL aqueous suspension of 100 nm SiO_2_ nanospheres for 5 min lead to formation of a nanosphere monolayer. After drying, 100 nm of silver was thermally deposited by the thermal evaporator (VRF-200M/ERH, Ulvac Kiko Inc., Miyazaki, Japan) at a typical deposition rate of 0.1 nm/s under a 3 × 10^−3^ Pa vacuum to prepare a standard SERS substrate. Alternatively, to prepare a patterned SERS substrate, the step of forming a nanosphere monolayer was followed by exposure of the glass slide to a 30 vol% decyltrimethoxy silane (DCTMS: Cat. No. LS-5258, Shin-Etsu Chemical Co., Ltd., Tokyo, Japan) ethanol solution for 15 min to make the surface superhydrophobic. Washing the glass slide with isopropyl alcohol (Cat. No. 164-08335, Wako Pure Chemical In., Ltd., Osaka, Japan) resulted in a significantly greater contact angle than with washing with ethanol or blow-drying. Finally, to make a SERS spot in the middle of the superhydrophobic area, 100 nm of silver was thermally deposited onto the glass slide through a pin hole metal mask with spot diameters of 0.4, 0.7, 1.0 or 1.3 mm. Blinking was often observed. Although the peaks of our interest in the current study were outside the region affected by blinking, we solved this problem by reducing the sensitivity of our substrates. They were heated to 60 °C for 48 h. This often eliminated the blinking problem.

SERS evaluation of the enzymatic reaction was carried out as follows. For the standard non-patterned SERS substrate, equal volumes (10 μL) of the AChE and ATC solutions were mixed and allowed to react for 5 min. If the effect of an inhibitor was to be monitored, equal volumes (5 μL) of the AChE and inhibitor solutions were mixed first and allowed to stand for 5 min, followed by addition of 5 μL of 10^−4^ M ATC solution. From the reactant mixture, 5 μL was placed on a standard non-patterned SERS substrate. After 2 min, the excess mixture was blow-dried with compressed air. For the evaluation of inhibitors with the patterned SERS substrate, after 5 μL of the ATC was added to the original mixture of the AChE and inhibitor as described immediately above, 1 μL of the final mixture consisting of AChE, inhibitor and ATC was placed on the patterned SERS substrate. It was then placed on a hot plate (AsOne Ceramic Hot Plate CHP170DF, AS ONE Co., Osaka, Japan) kept at 90 °C in order to rapidly terminate the enzymatic reaction and pre-concentrate the enzymatic end product, TC, onto the SERS spot. Salt crystals that would interfere with SERS measurements were gently washed off with water. For the evaluation of the uniformity of the SERS substrate, it was exposed to a saturated vapor of methyl mercaptan in a 1500-mL container for 15 min. The condition for obtaining a SERS spectrum with the Nicolet Almega XR, Thermo Fisher Scientific Raman spectrometer was as follows; an excitation wavelength of 633 nm at an output of 980 μW with a 1-sec acquisition time, averaged over 16 exposures. Spectra were obtained from five randomly selected spots to calculate the average and its standard deviation.

For contact angle measurements, 1 μL of distilled water was placed on surfaces to be evaluated; (a) a glass slide as delivered, (b) a glass slide treated with DCTMS, (c) a glass slide covered by a monolayer of silica nanospheres, (d) a glass slide covered by a monolayer of silica nanospheres and treated with DCTMS, and (e) the substrate in (d) covered by 100 nm of Ag. Photos of the 1 μL water droplet were taken with a Nikon Micro lens.

For assessing the extent of concentration and its dependence on the SERS spot diameter, lightly-colored water was prepared by mixing water color into distilled water. The purpose of using coloring was to facilitate the visualization of the extent of the dried spot. A small volume of the colored water was slowly injected onto a patterned SERS spot with aid of an electro-osmosis micropump (Nano Fusion Technologies, PS-24) until a droplet of the colored water was observed to just about completely cover the SERS spot underneath. A photo was taken to assess the volume of the droplet. The glass slide was placed on the hot plate to accelerate the drying of the droplet, after which it was checked to see whether residues from the colored water droplet remained within the SERS spot. Finally, the ratio between the initial volume and the SERS spot surface area was calculated to estimate the extent of pre-concentration.

As a conventional method for evaluating AChE activity, colorimetric assays based on the Ellman protocol were carried out as follows. ATC was diluted with water to prepare 10^−1^, 10^−2^, 10^−3^ and 10^−4^ M ATC solutions. 5,5′ dithiobis (DTNB), (Dojindo Laboratories Kumamoto, Japan, Cat. No. 69-78-3) was diluted with water to prepare a 0.01 M DTNB solution. AChE was diluted with 1 × Tris HCl buffer to prepare 5, 0.5, 0.05 and 0.005 U/mL AChE solutions. Three-hundred microliters each of the AChE enzyme and substrate solutions were mixed and allowed to react for 2 min. Three-hundred microliters of a 0.01 M DTNB solution was added to the above mixture solution and allowed to react for 1 min. The absorbance at a wavelength of 412 nm was measured with the Shimazu UV 1800 spectrophotometer. To observe the effect of heating on AChE, its solution was placed within a micro tube and submerged in a hot water bath at a preset temperature for five min.

## 4. Conclusions

We have shown that the random AgFON is a convenient SERS platform by virtue of the fact that the surface-adsorbed silica nanospheres can serve first as a rough surface suitable for the formation of a superhydrophobic surface upon DCTMS treatment and secondly as a SERS surface upon silver deposition. A pin hole mask used in conjunction with the latter process dictates the diameter of the SERS spot. This also allowed us to dry the sample completely, thus capturing all the target molecules on the surface. The SERS spot, which is incidentally less hydrophobic, can anchor an aqueous sample placed on it, significantly facilitating the transfer of a small-volume aqueous sample from a dispensing device to the SERS surface. After drying, target molecules were pre-concentrated for greater SERS signals. To the best of our knowledge, this is the first time that such an approach has been investigated. We evaluated its performance with a model compound, BPE, and confirmed that a three-orders of magnitude reduction in the required BPE concentration was possible while a similar 1010 cm^−1^ peak could be obtained. We also found that of the four SERS spot diameters we explored, 0.4, 0.7, 1.0 and 1.3 mm, there was relatively little dependence on it. Even though a geometrical consideration favored larger spot diameters, in reality SERS spots with smaller diameters were better able to confine the aqueous droplet, such that there was no clear advantage for using larger SERS spots. On the other hand, smaller SERS spots required more careful handling of small-volume sample, meaning that the spot size of 0.7 mm was found to be a good choice.

Furthermore, we tested its utility with a medically relevant system. AChE is an enzyme that plays an important role in nerve signal transmission. Use of SERS for detecting the end product, TC has already been demonstrated, but this may be the first time that surface-adsorbed silver nanostructure was used rather than colloidal solution. In comparison to the conventional colorimetric assay, a three-order of magnitude reduction in the sample volume was found to be possible while using AChE and ACT of comparable concentrations, 0.5 U/mL and 10^−4^ M, respectively. With the patterned SERS substrate, a significant reduction in the required AChE concentrations was achieved, from 0.05 to 5 × 10^−13^ U/mL. This was made possible through drying of the aqueous sample rather than blow-drying, but this in turn was made possible by the use of the patterned SERS substrate. Thus, capturing all TC produced from the enzymatic reaction led to this drastic reduction in the required AChE concentration. We also demonstrated that inhibitory effects of paraoxon and carbaryl could be readily monitored. Dependence of the TC characteristic peak at 768 cm^−1^ on the inhibitor concentration was greater for paraoxon than for carbaryl.

In this work, we described results from AChE experiments, but it is obvious that many other enzymes can be monitored in a similar fashion [51]. The key is finding proper enzyme substrates whose SERS activity can be switched on or “unmasked” upon the enzyme activity, and the end product exhibits a high affinity toward a noble metal surface [52,53].

## 5. Patents

H. Takei and R. Shitara have obtained the Japanese patent No. 5967756 for the patterned SERS substrate.

## Supplementary Materials

The following are available online. Figure S1: Procedure for preparing a patterned SERS substrate, Figure S2: Spectra of methyl mercaptan for evaluation of the uniformity of nanostructures, (a) spectra from five-randomly selected spots from a single replicate, (b) base-line adjusted 678 cm^−1^ peaks, (c) results from three separate replicates. Figure S3: Dependence of the 1010 cm^−1^ peak intensity on the effective concentration of BPE, Figure S4: The intensity of the 768 cm^−1^ peak, obtained with the standard SERS substrate, for ACT concentrations of 10^−1^, 10^−2^ and 10^−3^ M. The AChE concentration was varied in the range from 5 to 0.005 U/mL, Figure S5: AChE reaction evaluated by the conventional colorimetric assay with 10^−4^ M ATC and AChE in the range of 5 down to 0.005 U/mL. The required sample volume was 800 μL, Figure S6: Effects of elevating the temperature of AChE solution evaluated by SERS and colorimetric assays, Video 1: Attempt to place a 1 µL droplet of colored water onto a uniformly superhydrophobic region, Video 2: Attempt to place a 1 µL droplet of colored water onto a patterned SERS spot.
