# Supplementary material for: Patterned Superhydrophobic SERS Substrates for Sample Pre-Concentration and Demonstration of Its Utility through Monitoring of Inhibitory Effects of Paraoxon and Carbaryl on AChE
Source: Molecules. 2020 May 8;25(9):2223. doi: 10.3390/molecules25092223 (PMC7248789; doi:10.3390/molecules25092223)
Supplement: Supplementary file 1 [file molecules-25-02223-s001.pdf]

Figure S1

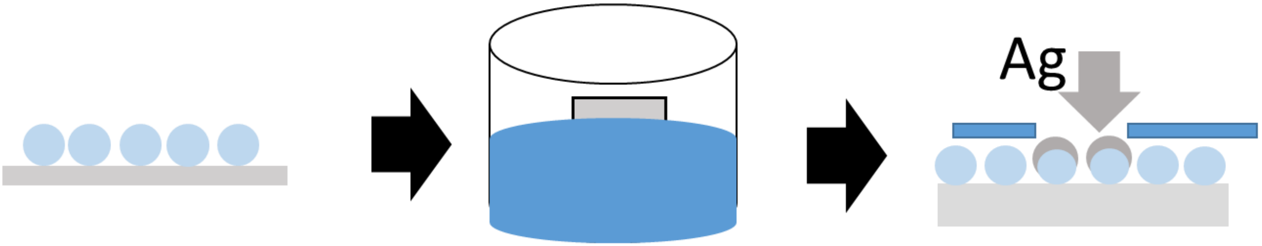

Silica nanospheres adsorbed on a glass slide

Immersed in a DCTMS solution

One-hundred nm of silver evaporated through a pin hole mask

DCTMS-modified SiO_2 nanospheres: superhydrophobic

Random AgFON: less hydrophobic

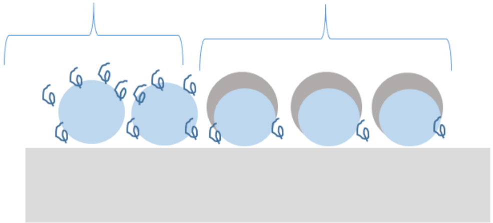

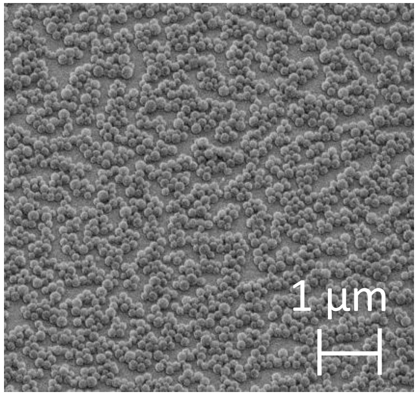

Figure S2

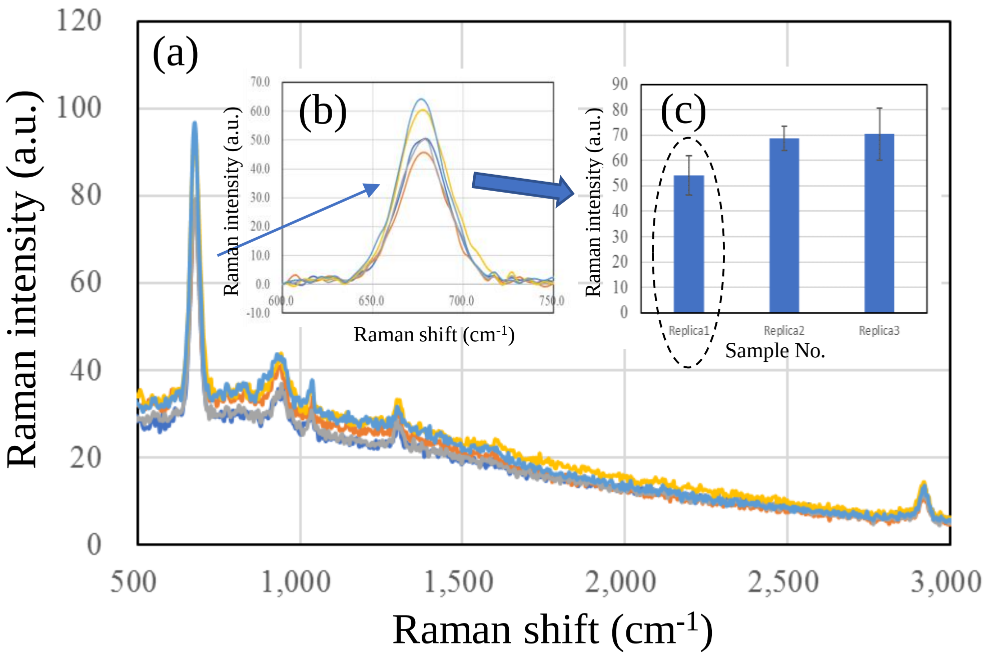

(a)
Raman intensity (a.u.)
120
100
80
60
40
20
0
500
1,000
1,500
2,000
2,500
3,000
Raman shift (cm-1)
(b)
Raman intensity (a.u.)
70.0
60.0
50.0
40.0
30.0
20.0
10.0
0.0
-10.0
600.0
650.0
700.0
750.0
Raman shift (cm-1)
(c)
Raman intensity (a.u.)
90
80
70
60
50
40
30
20
10
0
Replica1
Replica2
Replica3
Sample No.

Figure S3

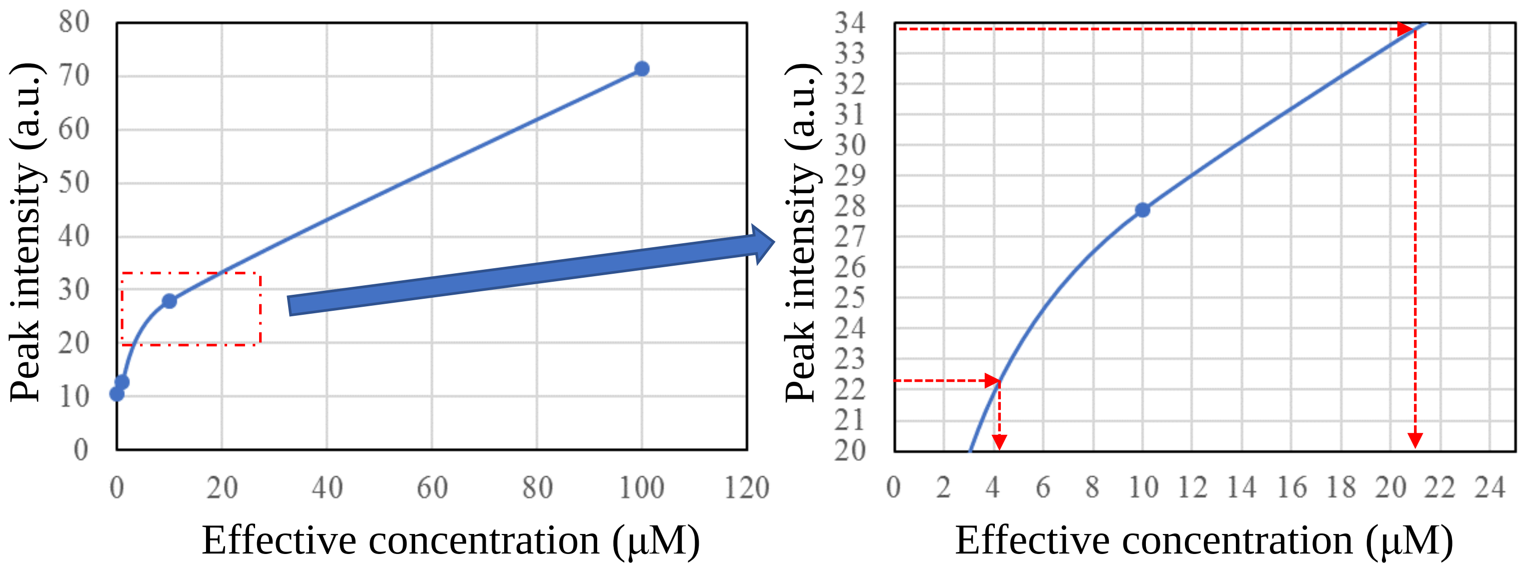
80
70
60
50
40
30
20
10
0
Peak intensity (a.u.)
0
20
40
60
80
100
120
Effective concentration (μM)
34
33
32
31
30
29
28
27
26
25
24
23
22
21
20
Peak intensity (a.u.)
0
2
4
6
8
10
12
14
16
18
20
22
24
Effective concentration (μM)

Figure S4

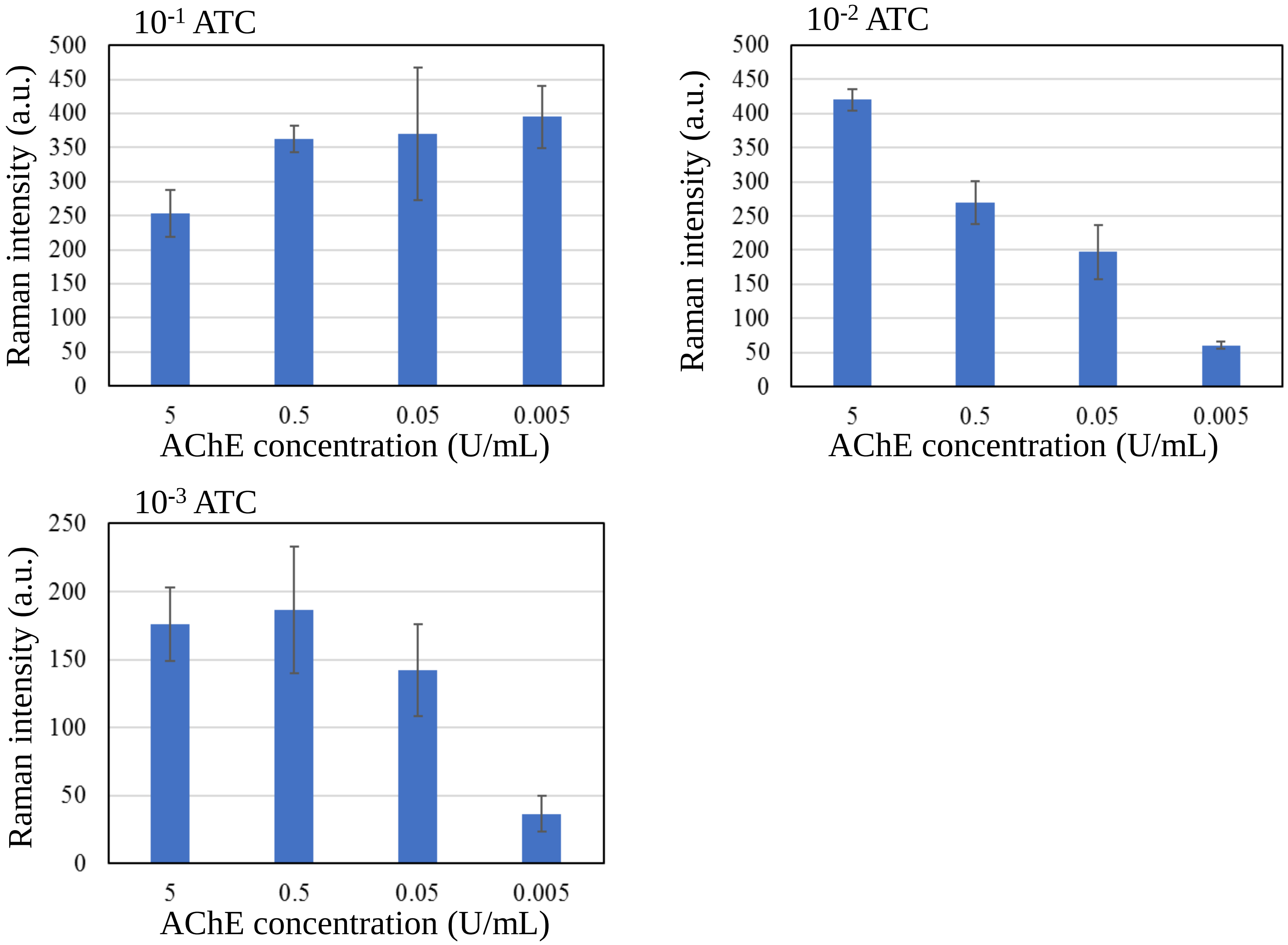
10-1 ATC
Raman intensity (a.u.)
500
450
400
350
300
250
200
150
100
50
0
5
0.5
0.05
0.005
AChE concentration (U/mL)
10-2 ATC
Raman intensity (a.u.)
500
450
400
350
300
250
200
150
100
50
0
5
0.5
0.05
0.005
AChE concentration (U/mL)
10-3 ATC
Raman intensity (a.u.)
250
200
150
100
50
0
5
0.5
0.05
0.005
AChE concentration (U/mL)

Figure S5

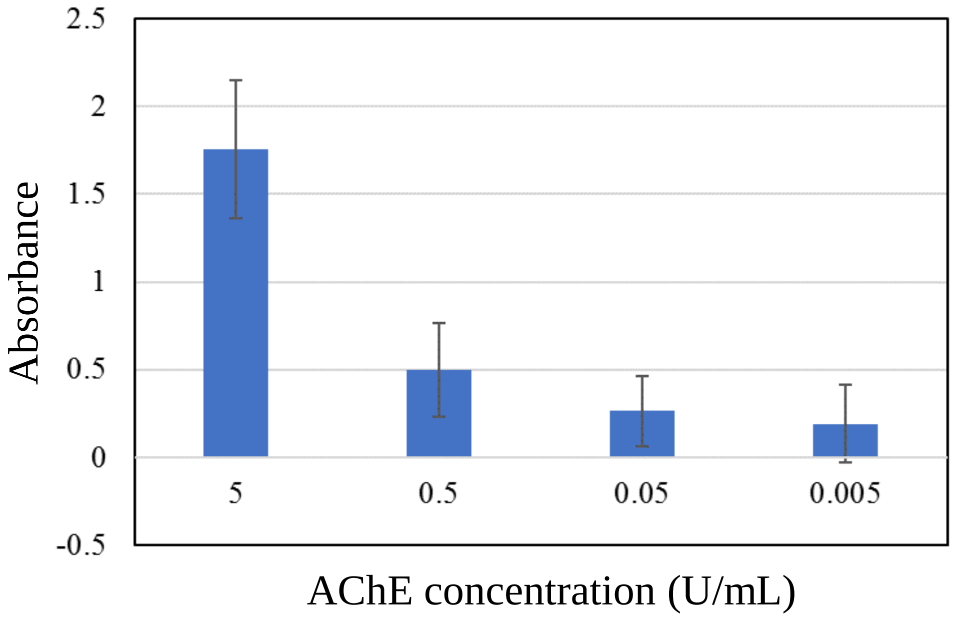

2.5
2
1.5
1
0.5
0
-0.5
Absorbance
5
0.5
0.05
0.005
AChE concentration (U/mL)

Figure S6

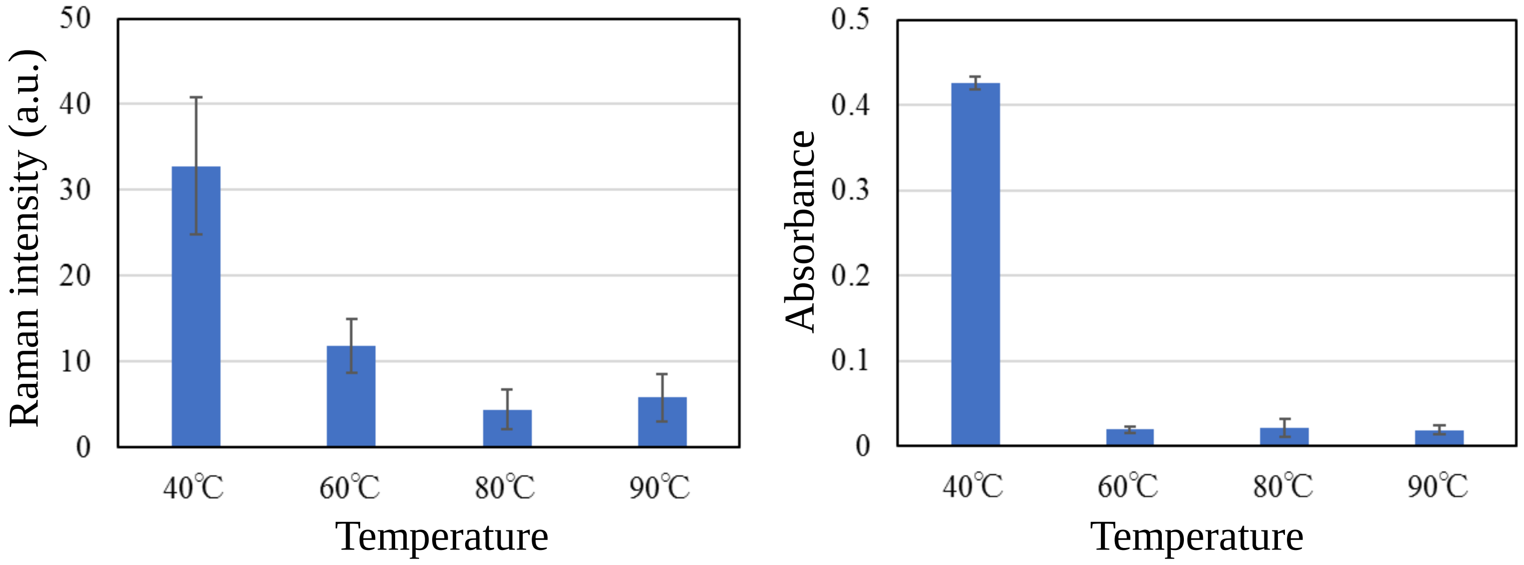

50
40
30
20
10
0
Raman intensity (a.u.)
40℃
60℃
80℃
90℃
Temperature
0.5
0.4
0.3
0.2
0.1
0
Absorbance
40℃
60℃
80℃
90℃
Temperature
